# Knowledge and Awareness Among Mothers Regarding Early Childhood Development: A Study From the United Arab Emirates

**DOI:** 10.7759/cureus.37027

**Published:** 2023-04-02

**Authors:** Sirine Saleh, Maitha AlGhfeli, Latifa Al Mansoori, Aysha Al Kaabi, Salwa Al Kaabi, Satish C Nair

**Affiliations:** 1 Academic Affairs, Tawam Hospital, Al Ain, ARE; 2 General Pediatrics, Tawam Hospital, Al Ain, ARE; 3 Department of Medical Affairs, The College of Medicine, Tawam Hospital, Al Ain, ARE

**Keywords:** awareness, parents, gulf, middle east, children

## Abstract

Background and objective

There is a dearth of studies on the knowledge and awareness among mothers on childhood development in the United Arab Emirates (UAE). Maternal knowledge of childhood development is a critical determinant of children's development and behavior. In light of this, we conducted this study to determine the level of maternal knowledge about childhood development.

Methodology

We employed a cross-sectional study design involving 200 mothers of all ages recruited using stratified random sampling. After obtaining informed consent, the participants were required to complete a questionnaire adapted from the "Ages and Stages" questionnaire, which covered demographics and items on developmental milestones. The questionnaire was validated and checked for reliability by using a focus group. Inferential statistics were used, and the association between the variables was analyzed using the Chi-squared test.

Results

Our findings indicated that the knowledge among mothers regarding child development is relatively low in the UAE. Two-thirds of the respondents were knowledgeable about gross motor skills (62% of the mothers knew the age at which a child could lift his/her head). Less than half of the mothers were well-informed about fine motor skills such as writing and drawing (44% of the mothers were aware of the age at which a child should be able to scribble on paper). The respondents demonstrated a lack of knowledge regarding children's speech and language skills. Regarding social skills, only 8% of the mothers were aware of the correct age at which a child should start to dress by himself/herself.

Conclusion

Although mothers in the UAE were knowledgeable about certain aspects of childhood development such as gross motor development, they were less knowledgeable regarding other aspects such as social and language skills. The gaps identified in our study highlight the need to implement effective health education programs so that mothers are better informed to help improve child development outcomes in the community.

## Introduction

It is well-known that awareness among mothers regarding early childhood development can contribute to a broad range of positive developmental effects [1]. Several initiatives have been undertaken to improve the developmental milestones in infants and young children, thanks to the increased awareness about infant brain development and its effects on later life [2,3]. Developmental milestones refer to the child's developmental stages that occur within a predictable age range, and include, but are not limited to, walking, talking, and feeding themselves. It can also be described as a set of skills that every child should acquire by a certain age [4]. The term developmental delay implies children failing to acquire these skills by an ascertained normal span for their age [4]. Furthermore, each individual develops at a unique rate, making it challenging to pinpoint exactly when a particular ability will be mastered. However, as a kid gets older, developmental milestones offer a broad approximation of when to notice specific changes. For instance, most babies start crawling at eight months and walking at 18 months of age. If a baby is four or five months behind in achieving these milestones, he/she is considered developmentally delayed [4]. There are four main domains in childhood development: physical, cognitive, language, and social-emotional. By monitoring these developmental milestones, mothers can obtain a general idea as to what to expect from their children in terms of their development [4]. These would help them be well-informed in terms of a child's developmental needs and consequently aware of when a child has not gained the developmental skills expected of him/her when compared to others of the same age. Delays may occur in the areas of motor function, speech and language, cognition, play, and social skills [5].

Parenting is a multifaceted endeavor and requires a systematic response to children's various needs. Parents should develop both depth and breadth of knowledge, ranging from being aware of developmental milestones and norms that help in keeping children safe and healthy to understanding the role of professionals (e.g., educators, childcare workers, healthcare providers, and social workers) and social systems (e.g., institutions, laws, and policies) that interact with families and support parenting. One focus of many initiatives to support parenting is on the significance of parents' understanding of child development. The need for policy and program initiatives to promote parenting knowledge is emphasized by evidence-based recommendations made by the American Psychological Association Task Force on Evidence-Based Practice with Children and Adolescents (2008), the Centers for Disease Control and Prevention (CDC) (2015), and the World Health Organization (WHO) (2009). Based on these guidelines, parents require a basic awareness of baby and child developmental milestones and norms as well as the kinds of parenting techniques that encourage children to attain these milestones to optimize their growth [6].

Studies conducted in developed countries have found that mothers' being knowledgeable about their children's developmental stages correlates positively with their ability to help them develop better [7]. Studies have shown that parents who do not know much about child development often overestimate or underestimate the ages at which children acquire certain skills. Sometimes these inaccurate estimates lead to unexpected outcomes for their kids' lives [8]. Studies from Western countries have shown that maternal knowledge of the development of their infants and young children has important implications. Clinicians often depend on mothers' knowledge of their child's health and development for decision-making, advice, and referrals [9].

The WHO Department of Child and Adolescent Health and Development and the United Nations Children's Fund (UNICEF) have jointly created the Care for Development Intervention (CDI) to assess the disparity between developed countries and third-world nations regarding the knowledge available for caregivers on how to support their child's development [10]. CDI imparts knowledge to medical professionals on how to perform a structured assessment to evaluate the ways the caregiver interacts and plays with the child and to give the caregiver suggestions for engaging the child in age-appropriate activities. CDI has been demonstrated to be a useful tool for assisting parents in their efforts to provide a more fulfilling environment for their children [11]. It has also been shown to be an effective method of supporting caregivers' efforts to ensure a more stimulating milieu for the children [11]. It was shown in a controlled trial that after a month of intervention, the families who received CDI counseling during one acute medical visit had considerably higher effective Home Observation for Measurement of the Environment scores, more home-made toys, and more children's books compared to control subjects who only received standard medical care. Despite the availability of numerous studies conducted in the Western setting, structured studies addressing maternal knowledge of a child's developmental milestones are scarce. This is particularly significant given the cultural, religious, economic, and psychosocial variability prevalent in a non-western setting. In light of this, the purpose of the current study was to assess the knowledge of mothers about their children's developmental milestones in the United Arab Emirates (UAE) by using a structured questionnaire.

## Materials and methods

This was a cross-sectional study conducted at a tertiary care center in Al Ain, the eastern region of the UAE. The questionnaire was framed by adopting questions from the "Ages and Stages" questionnaire 3rd edition [12] and included items on demographic variables and targeted questions about developmental milestones. The survey was made available in Arabic through a procedure that included cross-cultural verification, translation by the translation team, and additional revision by a skilled physician. The 15-item self-administrated survey measured parents' understanding of children's developmental landmarks in the four main domains: speech and language (four questions), fine motor (four questions), and gross motor (three questions), as well as 14 variables related to demographic factors, including the mother's age, educational attainment, marital status, citizenship, and country of origin, vocation, the total number of children, age of the youngest child, living arrangement (whether living with extended family or not), availability of a housemaid, and husband's age, occupation, and education level. One question was about the parents' source of information. The questionnaire was validated for the local setting by conducting a pilot study and subsequent validation by a focus group created for the purpose. To ensure the survey's validity and reliability, pilot research with 10 respondents was carried out. Assessment of mothers' knowledge about children's developmental milestones was the primary objective. The secondary objective was to determine the correlation between their knowledge and different demographic variables.

Data were collected by distributing the questionnaire to the participants living in different areas of the UAE, irrespective of age, by adopting the stratified random sampling method. An online survey through SurveyMonkey was sent anonymously to participants through emails and mobile applications, which included an explanation of the study and its purpose, with informed consent required before participation and completion of the questionnaire. The Institutional Review Board of Tawam Hospital approved the study protocol (MF2058-2020-759). Data were analyzed using IBM SPSS Statistics (IBM Corp., Armonk, NY). A p-value <0.05, was considered statistically significant. A Chi-squared test was performed to check the correlation between different variables.

## Results

The questionnaire was distributed to 240 mothers in various parts of the UAE, irrespective of their age. The response rate was 80% (200/240). The participants did not report any difficulty with understanding the survey items and the survey layout. The participant demographics are presented in Table 1. The highest proportion (30.6%) of participants were between the ages of 26-30 years; a significant majority (96.9%) of them were married, and 54.4% were university graduates. Approximately half of the participants (50.3%) were housewives, 78.2% had between one and four children, and 33.16% had their youngest child in the age group of one to three years. The majority of mothers (79.8%) had a housemaid for domestic help. The husbands of 28.5% of women were aged between 36 and 40 years; 45.24% of the husbands worked in the military, and 46.28% were college graduates. The mothers' major source of information about childhood development was their own experience (34.5%).

**Table 1:** Participant demographics (n=200)

Categories		Number (n)	Percent (%)
Age group, years	≤25	20	10%
	>40	26	13%
	26–30	59	31%
	31–35	47	24%
	36–40	41	21%
Education	Postgraduate degree	22	11%
	High school (grades 10–12)	38	20%
	Middle school (grades 7–9)	7	4%
	College graduate	20	10%
	University	105	54%
	None	1	1%
Marital status	Married	187	97%
	Single mother	4	2%
	Divorced	2	1%
Nationality	Non-Arab	11	6%
	Other Arab, Non-United Arab Emirates (UAE) national	32	17%
	UAE national	150	78%
Vocation	Student	12	6%
	Teacher	40	21%
	Healthcare provider	9	5%
	Housewife	97	50%
	Others	35	18%
Number of children	1–4	151	78%
	5–8	38	20%
	≥9	4	2%
Age of the youngest child, years	<1	55	29%
	>5	42	22%
	1–3	64	33%
	3–5	32	17%
Living with extended family	No	89	46%
	Yes	103	54%
Having a housemaid	No	39	20%
	Yes	154	80%
Husband’s age, years	≤25	4	2%
	>40	51	26%
	26–30	40	21%
	31–35	38	20%
	36–40	55	29%
	Not applicable (NA)	5	3%
Husband’s job	Healthcare provider	2	1%
	Teacher	5	3%
	Military	80	45%
	Administrator	20	10%
	Private sector employee	24	12%
	Others	69	34%
Husband’s education level	Elementary (grades 1–6)	6	3%
	Middle school (grades 7–9)	15	8%
	High school (grades 10–12)	57	30%
	College graduate	89	46%
	Postgraduate degree	23	12%
	Not applicable (NA)	2	1%
Source of information	Doctors or other healthcare providers	34	18%
	Internet	58	31%
	Social media	37	20%
	Personal experience	65	34%
	Media	32	17%

Table 2 shows the correct responses by the mothers to the questions in the questionnaire. The mothers were highly knowledgeable about gross motor skills, including the lifting of the head up (62%) and walking independently (63%); however, only 35% of them answered correctly regarding the exact age at which the child would sit up straight without support. In terms of fine motor skills, there was a moderate lack of knowledge. Only 40% of the mothers answered correctly regarding the exact age at which a child would be able to reach for a toy; 44% of them answered correctly regarding the exact age at which the child scribbles on a paper; 35% were aware of the age at which the child would start using a spoon for self-feeding; only 31% of the mothers were aware as to when to seek medical advice if the child did not transfer objects from one hand to the other. Additionally, mothers showed a significant lack of knowledge concerning the child's speech and language. Only 14% of them answered correctly regarding the age at which an infant would start cooing; 33% were aware of the correct age at which a child would say three words such as mama, baba, and dada; 46% were aware of the correct age at which a child would imitate a two-word sentence, and 40% of the mothers answered correctly about the age to seek medical advice in case the child did not use words at all. Mothers also showed a significant lack of knowledge about social skills. Half of them (49%) were aware of the age at which an infant would start smiling. However, only 8% were aware of the correct age at which a child would start dressing by himself/herself. Moreover, >52% of the mothers were aware of the age at which the child could perform activities of daily life such as brushing their teeth and hair, and only 20% were aware of the age at which to seek medical advice if the child did not maintain eye contact.

**Table 2:** Details of the correct responses from the participant mothers about developmental milestones

Items	Variables (all questions)	Correct responses (n)	Percent (%)
1	At what age do you think your infant/child can lift his/her head up when he/she is on the tummy for at least 15 seconds?	124	62
2	At what age do you think your infant/child should sit up straight for several minutes without support?	69	35
3	If your child did not walk independently, you will wait to seek medical advice until what age?	126	63
4	Fine motor: at what age do you think your infant/child will be able to reach for a toy?	80	40
5	At what age do you think your infant/child will be able to scribble on the paper?	88	44
6	At what age do you think your infant/child will start to use a spoon to feed him/herself?	69	35
7	If your child is not able to pass objects from hand to hand, you will wait to seek medical advice until what age?	62	31
8	Speech/language: at what age do you think your infant/child makes coo sounds like aah, gah, and ooo?	27	14
9	At what age do you think your infant/child says three words like mama, baba, and dada?	66	33
10	At what age do you think your infant/child imitates two-word sentences like "mama eat"?	91	46
11	If your child is not able to use words such as mama and papa/dada, you will wait to seek medical advice until what age?	79	40
12	Social: at what age do you think your infant/child will start smiling when you play with him/her (social smile)?	98	49
13	At what age do you think your infant/child helps dress him/herself?	15	8
14	At what age do you think your infant/child knows the function of daily living activities like (toothbrush and hairbrush)?	103	52
15	If your child lack or has limited eye contact, you will wait to seek medical advice until what age?	40	20

No statistically significant associations were found regarding mothers' knowledge about child development in terms of age, marital status, education, employment status, source of information, and husband's age, education, and career.

## Discussion

The study addresses the key issues related to major milestones in the development journey of children. Additionally, the study assessed the level of knowledge among mothers in the UAE about developmental milestones and examined its relationships with several factors, including parental age, educational level, employment status, parity, the age of the youngest child, source of information, nationality, and the use of childcare assistance. To the best of our knowledge, this is the first study to address mothers' knowledge about early developmental milestones in children in the UAE.

It is interesting to note that knowledge about gross motor development was adequate among parents. Typically, the advice that mothers receive from healthcare professionals is confined to the physical health of the child, with scant to no attention paid to the intellectual and psychological factors and parent-infant engagement abilities. Primary healthcare facilities naturally concentrate on monitoring a child's growth indicators, immunizations, and basic health-related issues such as eating, constipation, and nutritional disorders, and often fail to educate parents about anticipatory guidance, normal developmental milestones, and the red flags that should alert them of developmental delay. Our study indicated that the mothers possessed a fairly high level of knowledge regarding many of the physical aspects. This is consistent with a similar study from the Middle East, which demonstrated that mothers were more knowledgeable about physical and safety skills but less knowledgeable about cognitive, emotional, and parent-infant interaction skills [13].

In the present study, the mothers scored the least in the area of milestones for social skills, exemplified by the fact that only 8% of the mothers surveyed in our study correctly identified the age at which the infant or child could dress by himself/herself. This finding reflects the mothers' lack of knowledge regarding the social aspect of developmental milestones, which is in line with a previous regional study by Ibrahim HA in 2010. Similar to the findings reported by Rikhy et al., the knowledge of other domains, such as fine motor, language, and social competence, was comparatively poor among mothers [14]. The moms in our study exhibited significant gaps in terms of their knowledge about early developmental milestones, which could delay the recognition and detection of certain developmental abnormalities. Multiple variables had no impact on the mothers' understanding of parenthood, safety and health values, and development stages. There was no correlation between women's knowledge of parenting and their age, education, citizenship, occupation, the number of children, or spouses' age, education, or employment. These results are in line with those of several international studies, which revealed no association between parental age and children's physical, emotional, and cognitive development [15]. As reported in a previous study, older mothers have higher knowledge scores compared to adolescent mothers [16]. However, our study did not show any difference in knowledge between young and old mothers [16].

The present study has some limitations. Firstly, given the nature of the study design, no causation can be attributed to the observations. Additionally, the relatively small sample size means that the findings may not be representative of the general population. This also underlines the challenges faced in recruiting mothers willing to participate in the study. Regardless, the strengths of the study include the fact that this is the first study from the UAE to address the knowledge of mothers about their child's developmental milestones.

## Conclusions

Our study has illustrated significant gaps in the knowledge of mothers about their child's developmental milestones, especially in the social domain. The lack of parenting skills demonstrated may be partially attributed to the inadequacies in the engagement and interactions between healthcare professionals/caregivers and mothers. Nevertheless, nurses and physicians are mostly the first sources of knowledge about parenting for mothers. It is anticipated that the results of our study will be a stepping stone for researchers in countries with similar practices around the region. Further research that involves the participation of fathers and childcare providers should be conducted to attain a more comprehensive picture of factors affecting child developmental milestones in the UAE.
